# Lipid Classes and Fatty Acid Regiodistribution in Triacylglycerols of Seed Oils of Two *Sambucus* Species (*S. nigra* L. and *S. ebulus* L.)

**DOI:** 10.3390/molecules181011768

**Published:** 2013-09-25

**Authors:** Francisc Vasile Dulf, Ioan Oroian, Dan Cristian Vodnar, Carmen Socaciu, Adela Pintea

**Affiliations:** 1 Department of Environmental and Plant Protection, University of Agricultural Sciences and Veterinary Medicine Cluj-Napoca, Cluj-Napoca 400372, Manastur 3-5, Romania; E-Mails: francisc_dulf@yahoo.com (F.V.D.); neluoroian@yahoo.fr (I.O.); 2 Faculty of Food Science and Technology, University of Agricultural Sciences and Veterinary Medicine Cluj-Napoca, Cluj-Napoca 400372, Manastur 3-5, Romania; E-Mail: carmen.socaciu@usamvcluj.ro; 3 Faculty of Veterinary Medicine, University of Agricultural Sciences and Veterinary Medicine Cluj-Napoca, Cluj-Napoca 400372, Manastur 3-5, Romania; E-Mail: apintea@usamvcluj.ro

**Keywords:** *Sambucus* seed oils, fatty acid composition, lipid class, stereospecific analysis, GC-MS

## Abstract

The oil content and fatty acid composition of total lipids (TLs) and main lipid classes (NLs- neutral and PLs- polar lipids) in seeds of two wild *Sambucus* species (S. *nigra* and S. *ebulus*) from Transylvania (Romania) were determined by capillary gas chromatography (GC-MS). In addition, the positional distribution of fatty acids in seed triacylglycerols (TAGs) was determined by hydrolysis with pancreatic lipase. The seeds were found to be rich in fat (22.40–24.90 g/100g) with high amounts of polyunsaturated fatty acids (PUFAs) ranging from 68.96% (*S. ebulus*) to 75.15% (*S. nigra*). High ratios of PUFAs/SFAs (saturated fatty acids), ranging from 7.06 (S. *nigra*) to 7.64 (S. *ebulus*), and low ratios of n-6/n-3, ranging from 0.84 (S. *nigra*) to 1.51 (S. *ebulus*), were determined in both oils. The lipid classes/subclasses analyzed (PLs, MAGs—monoacylglycerols, DAGs—diacylglycerols, FFAs—free fatty acids, TAGs and SEs—sterol esters) were separated and identified using thin-layer chromatography. The fatty acid compositions of the TAG fractions were practically identical to the profiles of TLs, with the same dominating fatty acids in both analyzed species. SEs and FFAs, were characterized by high proportions of SFAs. The *sn*-2 position of TAGs was esterified predominantly with linoleic acid (43.56% for *S. nigra* and 50.41% for *S. ebulus*).

## 1. Introduction

In recent years, non-traditional vegetable oils have been used more and more in the healthcare industry due to their therapeutic properties [1]. These oils have become attractive from a nutritional standpoint, due to their unique phytochemical composition and antioxidant properties [2,3,4].

*Sambucus ebulus* L. (also called dwarf elder, elderberry or danewort) and *Sambucus nigra* L. (also known as black or European elderberry) are native perennial herbs of the Adoxaceae family in the order of the Dipsacales. The genus *Sambucus* grows in temperate to subtropical regions of the World. The plants tolerate relatively poor soil conditions and prefer the sunlight-exposed locations, but they can also grow in semi-shade situations [5]. *Sambucus nigra* L., due to their dark blue/purple fruits which are desirable to birds, rapidly colonize the areas along roadways, forest edges, and fence lines [6]. *Sambucus ebulus* L. is known in Romanian folk medicine mainly for its bacteriostatic and diuretic action [7]. In many countries of the World, leaves, flowers and berries of these plants are traditionally used for several medicinal applications [5,8]. The small, fully ripened fruits of *Sambucus* species are rarely used for fresh consumption, and they are mainly processed into jams, jellies and juices. Several *in vitro* studies indicate that these berries, due to their high content of anthocyanins and other polyphenolics, possess important antioxidant activity and anticarcinogenic, immune-stimulating, antibacterial, antiallergic, antiviral and anti-inflammatory properties [9,10,11,12,13].

The berry seeds are byproducts of the beverage and juice processing industry and their direct disposal in the environment can create serious environmental problems. Recent studies have shown that berry seed residues could be used as raw materials for the production of non-conventional seed oils with unique chemical properties and wide applications in the healthcare industry [1]. The role of dietary fats and oils in human nutrition is determined by their composition [4]. Moreover, the positional distribution of fatty acids in triacylglycerols could affect the nutritional value of lipids [14]. The available scientific reports on *Sambucus* berries refer to the composition of phenolic compounds [6,11,15] and their anti-oxidant properties [10,12], but few studies relate to lipid compositions [12,16,17,18,19]. The study of *Sambucus* fruit seed oils for their main lipid constituents (neutral and polar), may lead to value-added utilization of these fats and enhance the profitability of the fruit processing industries.

The objectives of this study were to compare the oil content and fatty acid composition of total lipids (TLs) and main lipid classes (polar lipids—PLs, monoacylglycerols—MAGs, diacylglycerols—DAGs, free fatty acids—FFAs, triacylglycerols—TAGs and sterol esters—SEs) in seeds of two wild *Sambucus* species (*S.* *nigra* and *S.* *ebulus*) growing on their natural sites in Transylvanian region (Romania). In addition, the positional distribution of fatty acids in seed TAGs was determined by enzymatic degradation with pancreatic lipase.

## 2. Results and Discussion

### 2.1. Oil Content of the Seeds

The data for the total seed lipid contents (expressed on the basis of seed dry weight) from wild berries of the *S. nigra* and *S. ebulus* are summarized in Table 1. There was no significant difference (*p* < 0.05) in oil yield of the analyzed seed species (24.90 g/100 g for *S. ebulus vs.* 22.40 g/100 g for *S. nigra* seeds). Very little data are available in order to compare the lipid contents of the wild fruit seeds studied in the present paper. Fazio *et al.* [12] examined the seed oil of *S. nigra* and measured an oil content of 1.59 g oil/10 g of dry seed flour. Helbig *et al.* [20] studied the health-beneficial ingredients remaining in the waste of various berry seeds and reported 12% of recovered oil from elderberry seed press residues. Johansson *et al.* [19] found that the seeds of the berry species belonging to genera *Vaccinium*, *Oxycoccus* and *Sambucus* were similar, having oil contents in a narrow range, from 24% to 33% on dry weight basis.

### 2.2. Fatty Acids Profile

The fatty acid compositions of TLs and lipid classes/subclasses, polar (PLs) and neutral (MAGs, DAGs, FFAs, TAGs, SEs), from seeds of two *Sambucus* species are listed in Table 1 and Table 2.

#### 2.2.1. Fatty Acid Composition in TLs

**Figure 1:**
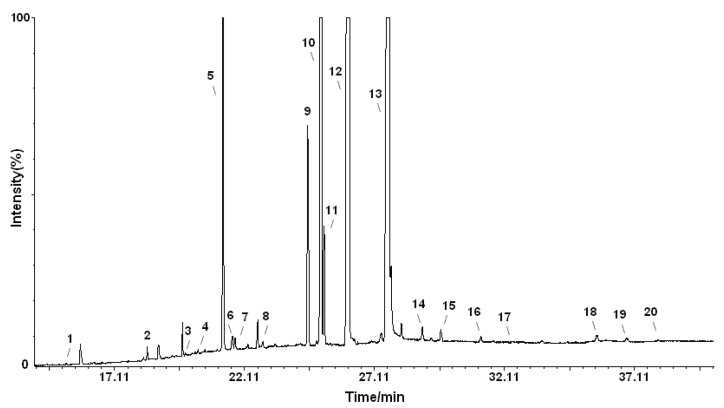
GC-MS chromatogram of FAMEs in the TLs of *Sambucus nigra* L. seeds analyzed with a SUPELCOWAX 10 capillary column.

According to the results shown in the Figure 1 and Table 1, twenty fatty acids were identified in both *Sambucus* seed oils. Comparing the TLs of two species, *S. ebulus* contained significantly higher proportion of oleic (C18:1n-9) and linoleic (C18:2n-6) acids (20.31% *vs.* 12.84%, *p* < 0.05 and 41.43% *vs.* 34.28%, *p* < 0.05, respectively) than *S. nigra*. The differences were greatest in the amounts of α-linolenic (C18:3n-3) (27.50% *vs.* 40.76%, *p* < 0.05) and palmitic (C16:0) (5.74% *vs.* 7.93%, *p* < 0.05) acids, but in the opposite direction. For *S. nigra* seed oils, Fazio *et al.* [12] reported a slightly lower α-linolenic acid content (approx. 32.10%) and similar values to those mentioned above for the other two major unsaturated fatty acids, linoleic (approx. 38.40%) and oleic (approx. 13.50%) acids, respectively.

Small amounts of stearic (C18:0) (<3%) and very small (<1.30%) (or trace) percentages of vaccenic (C18:1n-7), *cis*-7 hexadecenoic (C16:1n-9), arachidic (C20:0), 11-eicosenoic (C20:1n-9), palmitoleic (C16:1n-7), myristic (C14:0), margaric (C17:0), 11,14-eicosadienoic (C20:2n-6), behenic (C22:0), erucic (C22:1n-9), pentadecanoic (C15:0), azelaic, eicosatrienoic (C20:3n-3), tricosanoic (C23:0) and lauric (C12:0) acids were also determined in both *Sambucus* species (Table 1).

As shown in Table 2, statistically significant differences (*p* < 0.05) were found between fatty acid classes (excepting the very long chain saturated fatty acids fractions—VLCSFAs) of investigated berry seed TLs. The oil of both species contained high amounts of polyunsaturated fatty acids (PUFAs) ranging from 68.96% (*S. ebulus*) to 75.15% (*S. nigra*).

**Table 1:** Fatty acid composition (% of total fatty acids) of total lipid and individual lipid class.

	*Sambucus nigra* L.							*Sambucus ebulus* L.						
Fatty acids	TLs	PLs	MAGs	DAGs	FFAs	TAGs	SEs	TLs	PLs	MAGs	DAGs	FFAs	TAGs	SEs
12:0	0.01	0.20	0.06	0.17	0.43	0.02	1.27	tr.	0.06	0.07	0.35	0.51	0.02	0.58
14:0	0.09 ^a^	0.65	0.60	0.47	1.73	0.11	1.67	0.06 ^a^	0.23	0.20	0.72	0.96	0.08	1.06
15:0	0.02 ^a^	0.25	-	-	0.51	0.03	0.73	0.01 ^a^	0.09	0.07	0.28	0.43	0.02	0.46
AzA	0.02 ^a^	0.42	-	-	-	-	0.30	0.02 ^a^	0.17	0.01	0.20	-	-	-
16:0	7.93 ^a^	18.99	9.71	13.94	22.57	7.11	18.43	5.74 ^b^	15.22	8.77	9.60	15.78	5.80	9.99
16:1,n-9	0.15 ^a^	0.54	-	0.15	0.29	0.06	1.72	0.07 ^b^	0.19	0.29	0.76	0.69	0.05	0.92
16:1,n-7	0.08 ^a^	0.44	0.12	0.19	0.11	0.07	0.36	0.10 ^a^	0.12	0.11	0.40	0.18	0.09	0.17
17:0	0.04 ^a^	0.14	-	0.33	0.39	0.06	0.36	0.05 ^a^	0.14	0.06	0.07	0.25	0.04	0.09
18:0	2.29 ^b^	4.81	2.38	4.37	11.57	1.76	8.57	2.94 ^a^	5.72	3.43	5.42	8.73	2.39	5.54
18:1, n-9	12.84 ^b^	12.78	5.53	19.04	7.77	11.35	23.00	20.31 ^a^	20.40	22.62	31.29	19.62	20.85	16.82
18:1, n-7	0.94 ^b^	1.72	0.89	1.74	0.69	0.82	0.60	1.22 ^a^	1.79	2.53	2.02	1.31	1.11	0.92
18:2, n-6	34.28 ^b^	40.07	48.48	48.43	23.88	36.02	24.64	41.43 ^a^	40.58	47.24	31.40	28.73	45.03	36.15
19:0	-	0.10	-	-	-	-	0.33	-	0.05	-	-	0.06	-	-
18:3, n-3	40.76 ^a^	11.34	27.50	9.44	29.50	42.31	10.39	27.50 ^b^	13.16	13.19	12.82	19.00	24.21	19.02
20:0	0.15 ^a^	0.66	-	0.39	0.32	0.06	1.99	0.14 ^a^	0.66	0.14	0.38	0.73	0.06	0.32
2-OH-C16:0	-	0.63	-	-	-	-	-	-	0.20	-	-	-	-	-
20:1, n-9	0.14 ^b^	0.36	-	0.39	-	0.10	0.38	0.28 ^a^	0.34	0.39	0.74	0.39	0.17	0.49
20:2, n-6	0.07 ^a^	0.07	-	-	-	0.02	-	0.03 ^b^	-	0.06	-	-	0.01	-
20:3, n-3	0.04	-	-	-	-	0.06	-	tr.	-	-	-	-	tr.	-
22:0	0.08 ^a^	3.35	-	0.69	-	-	1.34	0.06 ^a^	0.18	0.02	0.18	0.98	-	0.17
22:1, n-9	0.06 ^a^	2.29	4.74	0.26	0.23	0.03	3.94	0.03 ^b^	0.59	0.81	3.37	1.37	0.08	7.28
23:0	0.01 ^a^	0.17	-	-	-	-	-	0.01 ^a^	0.10	-	-	0.27	-	-
*Oil content* *(g*/*100 g seeds)*				*22.40* *^a^*							*24.90* *^a^*			

The values represent the means of three samples, analyzed individually in triplicate (n = 3 × 3). TLs: total lipids, PLs: polar lipids, MAGs: monoacylglycerols, DAGs: diacylglycerols, FFAs: free fatty acids, TAGs: triacylglycerols, SEs: sterol esters, tr.: trace. Different superscript letters (^a,b^) in the same row mean significant differences between TLs of the two species (unpaired *t*-test). C12:0, lauric; C14:0, myristic; C15:0, pentadecanoic; AzA, azelaic; C16:0, palmitic; C16:1n-9, *cis*-7 hexadecenoic; C16:1n-7, palmitoleic; C17:0, margaric; C18:0, stearic; C18:1n-9, oleic; C18:1n-7,vaccenic; C18:2n-6, linoleic; C19:0, nonadecanoic; C18:3n-3, α-linolenic; C20:0, arachidic; 2-OH-C16:0, 2-hydroxy palmitic; C20:1n-9, 11-eicosenoic; C20:2n-6, 11,14-eicosadienoic; C20:3n-3, eicosatrienoic; C22:0, behenic; C22:1n-9, erucic; C23:0, tricosanoic acids.

**Table 2:** The composition (%) of fatty acid classes in total lipids and major lipid fractions.

	Fatty acids (% of total fatty acids)					
Species	∑ SFAs	∑ MUFAs	∑ PUFAs	∑ VLCSFAs (≥20C)	n-6/n-3	PUFAs/SFAs
**S.nigra**						
TLs	_a_10.64 ± 0.45^c^_de_	_b_14.21 ± 0.55^b^_d_	_a_75.15 ± 1.65^a^_a_	_a_0.25 ± 0.05^d^_de_	_b_0.84_e_	_a_7.06_b_
PLs	29.75 ± 1.15^b^_b_	18.14 ± 0.75^c^_c_	51.49 ± 1.25^a^_c_	4.18 ± 0.14^d^_a_	3.54_b_	1.73_e_
MAGs	12.75 ± 0.47^b^_d_	11.28 ± 0.38^b^_e_	75.97 ± 1.50^a^_a_	-	1.76_d_	5.96_c_
DAGs	20.36 ± 0.95^b^_c_	21.77 ± 0.95^b^_b_	57.87 ± 1.30^a^_b_	1.08 ± 0.12^c^_c_	5.13_a_	2.84_d_
FFAs	37.53 ± 1.35^b^_a_	9.09 ± 0.35^c^_f_	53.38 ± 1.22^a^_c_	0.32 ± 0.08^d^_d_	0.81_e_	1.42_f_
TAGs	9.15 ± 0.38^c^_e_	12.44 ± 0.50^b^_de_	78.42 ± 1.70^a^_a_	0.06 ± 0.02^d^_e_	0.85_e_	8.57_a_
SEs	34.98 ± 1.20^a^_a_	30.00 ± 1.10^b^_a_	35.02 ± 1.30^a^_d_	3.33 ± 0.12^c^_b_	2.37_c_	1.00_g_
**S. ebulus**						
TLs	_b_9.02 ± 0.40^c^_e_	_a_22.02 ± 0.80^b^_c_	_b_68.96 ± 1.55^a^_a_	_a_0.20 ± 0.05^d^_d_	_a_1.51_e_	_a_7.64_b_
PLs	22.62 ± 0.78^b^_b_	23.44 ± 0.75^b^_c_	53.74 ± 1.30^a^_c_	0.94 ± 0.10^c^_b_	3.08_b_	2.38_e_
MAGs	12.77 ± 0.35^c^_d_	26.75 ± 0.72^b^_b_	60.49 ± 1.60^a^_b_	0.16 ± 0.04^d^_d_	3.59_a_	4.74_c_
DAGs	17.20 ± 0.72^c^_c_	38.57 ± 1.30^b^_a_	44.22 ± 1.22^a^_d_	0.57 ± 0.05^d^_c_	2.45_c_	2.57_e_
FFAs	28.70 ± 1.10^b^_a_	23.57 ± 0.70^c^_c_	47.73 ± 1.26^a^_d_	1.97 ± 0.15^d^_a_	1.51_e_	1.66_f_
TAGs	8.40 ± 0.35^c^_e_	22.35 ± 0.75^b^_c_	69.25 ± 1.60^a^_a_	0.06 ± 0.03^d^_d_	1.86_d_	8.24_a_
SEs	18.22 ± 0.75^c^_c_	26.60 ± 0.70^b^_b_	55.17 ± 1.35^a^_c_	0.49 ± 0.04^d^_c_	1.90_d_	3.03_d_

Values are mean *±* SD of three samples, analyzed individually in triplicate (n = 3 × 3). Different subscript letters (_a, b_) in front of the mean values in the same columns indicate significant differences (*p* *<* 0.05) between total lipids (TLs) of two analyzed seed species (unpaired *t*-test).; Means in the same row followed by different superscript letters (^a, b, c, d^) indicate significant differences (*p* *<* 0.05) among fatty acid classes; Means in the same column followed by different subscript letters(_a, b, c, d, e, f_) indicate significant differences (*p* *<* 0.05) among lipid classes of each seed sample (ANOVA “Tukey’s Multiple Comparison Test”); SFAs—saturated fatty acids, MUFAs- monounsaturated fatty acids, PUFAs-polyunsaturated fatty acids, VLCSFAs—very long chain saturated fatty acids; TLs—total lipids, PLs—polar lipids, MAGs—monoacylglycerols, DAGs—diacylglycerols, FFAs—free fatty acids, TAGs—triacylglycerols, SEs—sterol esters.

Peaks: (1) C12:0, lauric; (2) C14:0, myristic; (3) C15:0, pentadecanoic; (4) AzA, azelaic; (5) C16:0, palmitic; (6) C16:1n-9, *cis*-7 hexadecenoic; (7) C16:1n-7, palmitoleic; (8) C17:0, margaric; (9) C18:0, stearic; (10) C18:1n-9, oleic; (11) C18:1n-7,vaccenic; (12) C18:2n-6, linoleic; (13) C18:3n-3, α-linolenic; (14) C20:0, arachidic; (15) C20:1n-9, 11-eicosenoic; (16) C20:2n-6, 11,14-eicosadienoic; (17) C20:3n-3, eicosatrienoic; (18) C22:0, behenic; (19) C22:1n-9, erucic; (20) C23:0, tricosanoic acids.

These levels are comparable to those of PUFA-rich vegetable oils, such as grape seed (65.40%), sunflower (66%), paprika seed (67.80%), perilla (69.90%), linseed (71.80%), blackcurrant seed (75.30%), safflower (77.30%) and hemp seed (79.10%) oils [21]. Moreover, the present analysis indicated that PUFAs profile of *S. nigra* seed oil resembles the seed oils of Ericaceae and sea buckthorn berries, whereas the proportion of oleic (C18:1n-9), linoleic (C18:2n-6) and α-linolenic (C18:3n-3) acids in TL fraction of *S. ebulus* seeds are similar to those of some seed oils of Rosaceae berries [19].

The oils of both species were characterized by high ratios of PUFAs/ saturated fatty acids (SFAs) (Table 2). These types of vegetable oils are susceptible to oxidative damage because of their high content in linoleic and α-linolenic acids [22]. The ratio of n-6 to n-3 fatty acids in TL of *S. ebulus* (1.51) was significantly higher (*p* < 0.05) than that in *S. nigra* TL (0.84). Epidemiological and clinical studies suggest that lowering the dietary n-6/n-3 fatty acid ratio may reduce the risk of coronary heart disease and cancer [23,24].

#### 2.2.2. Fatty Acid Composition of TAGs

The data for the fatty acid compositions of TAGs in the seeds of two *Sambucus* species are shown in Table 1 and Table 2. The fatty acid patterns of the TAG fractions (the major neutral lipid class of seed oils) were practically identical to the profiles of TLs, with the same dominating fatty acids in both analyzed species.

Comparing data from the present study with those reported by Johansson *et al.* [19] for the main fatty acids from seed oil TAG of *Sambucus racemosa*, important differences in SFAs, MUFAs (monounsaturated fatty acids) and PUFAs contents were observed. However, the linoleic acid amount from *S. ebulus* seed oil TAG (45.03%) is similar to that determined in seed oil TAG of *Sambucus racemosa* (46.10%). The geographical and climatic conditions, as well as cultivating activities, genetic differences among species, maturity stages of the seeds and lipid extraction methods could explain these differences in fatty acid compositions [25,26].

#### 2.2.3. Fatty Acid Composition of Minor Neutral Lipid (NL) Subclasses

The NL fraction of vegetable oils mainly consists of TAGs. However, small amounts of partial glycerides (MAGs and DAGs) and FFAs are always present, whose origin could be traced to biosynthetic and lipolytic (enzymatic or chemical) processes. All minor NL subclasses (MAGs, DAGs, FFAs and SEs) were highly unsaturated (Table 1 and Table 2).

The contents of unsaturated fatty acids (MUFAs+PUFAs), which mainly consisted of oleic, linoleic and α-linolenic acids (Table 1), ranged from 62.47% (in FFAs) to 87.25% (in MAGs) for *S. nigra* and 71.30% (in FFAs) to 87.24% (in MAGs) for *S. ebulus* (Table 2).

The levels of SFAs in SEs (34.98%—*S. nigra* and 18.22%—*S. ebulus*) and FFAs (37.53%—*S. nigra* and 28.70%—*S. ebulus*) of both seed oil were significantly higher (*p* < 0.05) than in the other two NL fractions, due to the dominance of palmitic acid in their structures (Table 1). It is interesting to note that in SE fraction of *S. nigra*, the VLCSFAs, namely arachidic and behenic, were estimated in a relatively low but significant amount and comprised about 3.30% of total fatty acids. In higher plants, VLCSFAs (with more than 18 carbons) are essential structural components of plant cuticular lipids [27,28].

As shown in Table 2, in SE and FFA fractions of *S. nigra* seed oil, the PUFAs/SFAs ratios were significantly lower (*p* < 0.05) (below 1.50) than in the other two corresponding NL subclasses (MAG and DAG ). Previous studies have shown that the values of this ratio comprised between 1.0 and 1.5, are optimal to reduce the risk of cardiovascular diseases [29,30].

#### 2.2.4. Fatty Acid Composition of PLs

The major PUFAs in PLs of *Sambucus* seed oils were linoleic, and α-linolenic acids, together comprising more than 50% of the total fatty acids (Table 1). Comparing with the other lipid fractions, the PLs, similar to SEs and FFAs, were characterized by high percentages of SFAs (*p* < 0.05). The amounts of saturated (consisting mainly of palmitic, stearic and VLCS fatty acids) accounted for 29.75% and 22.62% of total fatty acids in S*. nigra* oil PL and S*. ebulus* oil PL, respectively (Table 2). These observations are in accordance with those of our previous studies [26,28] and with the data reported by Zlatanov [31], Kallio *et al.* [25], Yang *et al.* [32], Gutierrez *et al.* [22] and Ramadan *et al.* [1], regarding to the fatty acid composition of the PL, SE and FFA fractions of other non-conventional seed oils.

The tested PLs had n-6 PUFAs to n-3 PUFAs ratios of 3.54 for *S. nigra* and 3.08 for S*. ebulus*. Very small amounts (<1%) of 2-hydroxy palmitic acid (2-OH-C16:0) were found in both PL fractions. These types of hydroxy fatty acids with important physicochemical and physiological properties in eukaryotic cells are synthesized by a sphingolipid fatty acid 2-hydroxylase, and are predominantly present in complex sphingolipids such as glucosylceramides and glycosylinositolphosphorylceramides [33]. In a recent study Herrero *et al.* [34] reported that 2-hydroxylated fatty acid-containing ceramides are involved in the mechanism of action of a novel synthetic antitumor drug (PM02734).

Differences between the fatty acid compositions of the studied lipid fractions could be attributed to the different phases of biosynthesis and accumulation of TAGs, SEs, PLs and fatty acids [35,36]. Studies have shown that the membrane phospholipids are more labile to oxidation than emulsified triacylglycerols. However, when these polar lipids are in an oil phase, they are more stable to oxidation than the triacylglycerols or free fatty acids [37,38].

#### 2.2.5. Positional Distribution of Fatty Acids in Seed TAGs

The positional distribution of fatty acids in *Sambucus* seed oils TAGs is shown in Table 3. The fatty acid composition of *sn*-1, 3 and *sn*-2 positions exhibited the similar patterns to the the total fatty acid composition of TAGs or TLs (Table 1). At all positions, the palmitic, oleic, linoleic and α-linolenic acids were the major fatty acids and together comprised more than 95% of total fatty acids (Table 3).The *sn*-2 position was esterified predominantly with linoleic acid: 43.56% in *S. nigra* seed oil *vs.* 50.41% in *S. ebulus* seed oil. Stearic, palmitic and α-linolenic acids were distributed primarily in the *sn*-1, -3 positions, while oleic acid was found in greater amount at the *sn*-2 position (Table 3). These are partially in accordance with the findings of Gunstone *et al.* [39]. Using an enzymatic method, these authors investigated the distribution of unsaturated fatty acids in vegetable TAGs and observed that linoleic acid was preferentially located in the secondary position, whereas oleic and α-linolenic acids were equally distributed in *sn*-1,-2, and -3 positions.

**Table 3:** Positional distribution (% of total fatty acids) of fatty acids in TAGs of *Sambucus* seed oil samples analyzed.

	*Sambucus nigra* L.		*Sambucus ebulus* L.	
	*sn*-position		*sn*-position	
Fatty acids (%)	*sn*-1,3	*sn*-2	*sn*-1,3	*sn*-2
(12:0)	0.03	0.01	0.04	0.01
(14:0)	0.11	0.05	0.11	0.06
(15:0)	0.03	-	0.03	0.02
(16:0)	8.57	0.97	7.78	1.20
(16:1,n-9)	0.06	0.05	0.09	0.10
(16:1,n-7)	0.06	0.05	0.09	0.10
(17:0)	0.04	-	0.06	-
(18:0)	1.80	0.60	3.06	0.94
(18:1, n-9)	8.71	16.79	15.33	29.31
(18:1, n-7)	0.99	0.24	1.50	0.54
(18:2, n-6)	32.59	43.56	42.88	50.41
(18:3, n-3)	46.77	37.62	28.76	17.21
(20:0)	0.06	-	0.06	-
(20:1, n-9)	0.08	-	0.17	-
(20:2, n-6)	0.07	-	0.03	-
(20:3, n-3)	0.04	-	tr.	-
(22:1, n-9)	-	0.06	-	0.10

Results are given as the average of triplicate determinations.C12:0, lauric; C14:0, myristic; C15:0, pentadecanoic; C16:0, palmitic; C16:1n-9, *cis*-7 hexadecenoic; C16:1n-7, palmitoleic; C17:0, margaric; C18:0, stearic; C18:1n-9, oleic; C18:1n-7, vaccenic; C18:2n-6, linoleic; C18:3n-3, α-linolenic; C20:0, arachidic; C20:1n-9, 11-eicosenoic; C20:2n-6, 11,14-eicosadienoic; C20:3n-3, eicosatrienoic; C22:1n-9, erucic.

**Figure 2:**
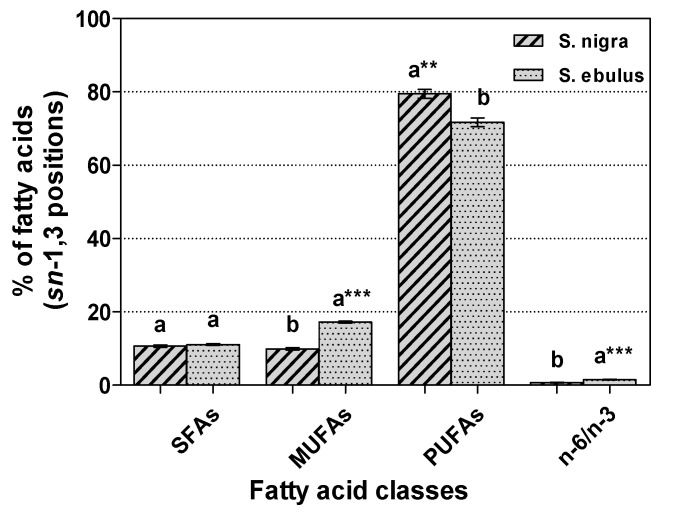
Positional distribution (%, means ± standard deviation, n = 3 × 3) of fatty acid classes in the TAGs *sn*-1, 3 positions of *Sambucus* seed oils.

The distribution patterns in *sn*-1,3 positions (Figure 2) were characterized by a higher percentage (*p* < 0.01) of PUFAs in *S. nigra* (79.46%) than in *S. ebulus* (71.67%). The difference was greatest in the proportions of MUFAs (9.89% *vs*. 17.19%, *p* < 0.001), but in the opposite direction. The ratio of n-6 to n-3 PUFAs was higher (*p* < 0.001) in *S. ebulus* (1.49) than in *S. nigra* (0.70). No significant difference (*p* > 0.05) was observed in the proportions of SFAs from the primary positions (10.64% in *S. nigra* and 11.14% in *S. ebulus*).

**Figure 3:**
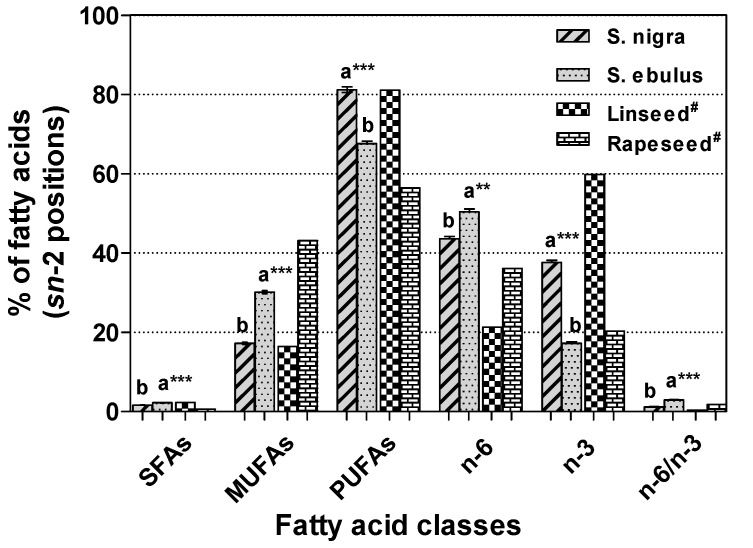
The percentage composition (means ± standard deviation, n = 3 × 3) of fatty acid classes in the TAGs *sn*-2 position of *Sambucus* seed, linseed and rapeseed oils.

^#^ Reference [40]. Different letters (a,b) in the same group mean significant differences, ** *p* < 0.01, *** *p* < 0.001 (unpaired *t*-test). SFAs—saturated fatty acids, MUFAs—monounsaturated fatty acids, PUFAs—polyunsaturated fatty acids, n-6—(n-6 PUFAs), n-3—(n-3 PUFAs).

In the case of *sn*-2 position of *Sambucus* seed oils TAGs (Figure 3), the levels of SFAs (2.23% *vs.* 1.63%, *p* < 0.001) and MUFAs (30.15% *vs*. 17.19%, *p* < 0.001) were significantly higher in *S. ebulus* than in *S. nigra*, and *vice versa* in PUFAs (67.62% *vs*. 81.18%, *p* < 0.001). It is generally known that unsaturated fatty acids are preferentially located in the *sn*-2 position and saturated fatty acids are distributed in *sn*-1 and *sn*-3 positions in TAGs of most vegetable oils [40,41]. The ratio of n-6 to n-3 PUFAs in the second position of *S. ebulus* (2.93) was significantly higher (*p* < 0.05) than that in *S. nigra* (1.16). These n-6/n-3 ratio values are close to the values recommended by Simopoulos [42] (n-6/n-3 = 1-5/1) as beneficial for good health.

Different letters (a,b) in the same group mean significant differences, ** *p* < 0.01, *** *p* < 0.001(unpaired *t*-test). SFAs—saturated fatty acids, MUFAs—monounsaturated fatty acids, PUFAs—polyunsaturated fatty acids, n-6/n-3—n-6 PUFAs to n-3 PUFAs ratios.

The n-3 PUFAs contents in *sn*-2 position of *S. nigra* (37.62%) and *S. ebulus* (17.21%) TAGs, due to their high content of α-linolenic acid, are much greater than that of most other plant oils, such as corn oil (0.7%), olive oil (0.8%) or soybean oil (7.1%), but comparable to that of rapeseed (20.3%) and linseed (59.8%) oils (Figure 3) [40]. For this reason, the *Sambucus* seed oils analyzed are optimal as food ingredients or food supplements to increase the intake of n-3 PUFAs. The α-linolenic acid is a precursor for the synthesis of longer chain n-3 PUFAs, such as eicosapentaenoic acid (20:5, n-3) and docosapentaenoic acid (22:5, n-3), which can promote visual, neural and vascular health [43]. Many studies have reported that the fatty acids located in *sn*-1, 3 and *sn*-2 positions of TAG have different metabolic fates in the human body [14,44,45,46]. It was observed that fatty acids in the *sn*-2 position of TAG are directly absorbed by the intestine, whereas those of the primary positions are released before absorption.

## 3. Experimental

### 3.1. Samples and Chemicals

The ripe berries of *S. nigra* and *S. ebulus* were collected from different parts of wild bushes on slopes of the Carpathian Mountains (northwest of Transylvania, Romania). The fruits were collected during September to October of 2012 at the stage of commercial maturity and were identified with the help of experts from the Department of Environmental and Plant Protection, University of Agricultural Sciences and Veterinary Medicine Cluj-Napoca, Romania. Berries were stored in polyethylene bags at −20 °C until analysis. Seeds were isolated manually from frozen berries, water-washed, and dried at 40 °C to a moisture content of about 7% in an air-drifted oven.

The lipid standards (used for identification of the lipid class) and chemicals [used for the total fat extraction, fractionation, enzymatic reaction and preparation of fatty acid methyl esters (FAMEs)] were of analytical grade (Sigma–Aldrich, St. Louis, MO, USA). Lipase from porcine pancreas (L-3126; Type II, 100–400 units/mg protein) was also purchased from Sigma-Aldrich. The thin layer chromatography (TLC) plates (silica gel 60 F254, 20 × 20 cm) were purchased from Merck (Darmstadt, Germany). The fatty acid methyl esters (FAMEs) standard (37 component FAME Mix, SUPELCO, catalog No: 47885-U) were obtained from Supelco (Bellefonte, PA, USA).

### 3.2. Extraction of Lipids

The TLs of the seeds were extracted using a chloroform/methanol mixture [26,47]. The sample (5g seeds) was homogenized in methanol (50 mL) for 1 min with a high-power homogeniser (MICCRA D-9, ART Prozess- und Labortechnik, Müllheim, Germany), then chloroform (100 mL) was added, and homogenization continued for 2 min. The mixture was filtered and the solid residue was resuspended in chloroform: methanol mixture (2:1, v/v, 150 mL) and homogenized again for 3 min. The mixture was filtered, and the residue was washed with chloroform: methanol (2:1, v/v, 150 mL). The filtrates and washings were combined and cleaned with 0.88% aqueous potassium chloride followed by methanol: water (1:1, v/v) solution. The purified lipid (bottom) layer was filtered and dried over anhydrous sodium sulfate and the solvent was removed in a rotary evaporator. The amount of lipids was noted. The recovered oils were transferred to vials with 4 mL chloroform (stock solution), and stored at −18 °C for further analysis.

### 3.3. Fractionation of TLs

Neutral (TAGs, MAGs, DAGs, FFAs and SEs) and PL fractions were separated by preparative TLC [28]. Aliquots of TL stock solutions (0.2 mL) were applied on the TLC plates, developed with petroleum ether: diethyl ether: acetic acid (80:20:2, v/v/v), sprayed with 2′,7′-dichlorofluoroscein/ methanol (0.1% w/v) and visualized under UV light (254 nm) [48]. The lipid classes/subclasses were identified using commercial standards, which were run in parallel with the samples. The separated PLs, MAGs, DAGs and FFAs bands were scraped off, and extracted with a mixture of chloroform/methanol (2:1, v/v). The bands corresponding to TAGs and SEs were also scraped off and extracted with chloroform. After samples were filtered, the solvent was removed and the dry residue was subjected to transesterification and gas chromatographic (GC) analysis.

### 3.4. Positional Fatty Acid Composition

TAGs were purified from total lipid extracts by preparative TLC using the conditions described above for resolution of NLs and PLs. Pancreatic lipase was used to generate *sn*-2-MAGs from TAGs [46,49]. Five milligrams of purified TAGs were placed in a test tube (previously equilibrated to 40 °C) and mixed with Tris-HC1 buffer (1M; pH 7.6, 5 mL), 0.05% (w/v) bile salt solution (1.25 mL), aqueous calcium chloride solution (2.2%, w/v, 0.5 mL), and pancreatic lipase (5 mg). The mixture was vortexed for 1 min, and incubated in a water bath at 40 °C for 3 min. The enzymatic reaction was stopped by adding ethanol (1 mL) followed by HCl (6 M, 1 mL). The hydrolysis products were extracted from the reaction medium with diethyl ether (2 × 2 mL) and separated on TLC plates using a solvent system of petroleum ether: diethyl ether: acetic acid (70:30:1, v/v/v). Bands that co-migrated with MAGs and DAGs standards were scratched, extracted with chloroform: methanol (2:1, v/v) and subjected to GC analysis (after transesterification).

### 3.5. GC Analysis of FAMEs

Fatty acids of TL, NL, PL, MAG (*sn*-2) and DAG (*sn*-1, 3) fractions were derivatized by acid-catalyzed transesterification procedure [28,50]. The FAMEs were determined by gas chromatography-mass spectrometry (GC-MS), using a PerkinElmer Clarus 600 T GC-MS (PerkinElmer, Inc., Shelton, CT, USA) [28]. The initial oven temperature was 140 °C, programmed by 7 °C/min until 220 °C and kept 23 min at this temperature. The flow rate of the carrier gas (helium) was 0.8 mL/min and the split value was a ratio of 1:24. A sample of 0.5 μL was injected on a 60 m × 0.25 mm i.d., 0.25 μm film thickness SUPELCOWAX 10 (Supelco Inc.) capillary column. The injector temperature was set at 210 °C. The positive ion electron impact (EI) mass spectra was recorded at an ionization energy of 70 eV and a trap current of 100 μA with a source temperature of 150 °C. The mass scans were performed within the range of *m/z*: 22–395 at a rate of 0.14 scan/s with an intermediate time of 0.02 s between the scans. Identification of FAMEs was achieved by comparing their retention times with those of known standards (37component FAME Mix, SUPELCO # 47885-U) and the resulting mass spectra to those in our database (NIST MS Search 2.0). The amount of fatty acids was expressed as percent of total fatty acids.

### 3.6. Statistics

Three different samples of *Sambucus* seeds for each species were assayed. The analytical results reported for the fatty acid compositions, are the average of triplicate measurements of three independent oils (n = 3 × 3). Statistical differences among samples were estimated using Student’s t-test and ANOVA (one-way analysis of variance; Tukey’s Multiple Comparison Test; GraphPad Prism Version 4.0, Graph Pad Software Inc., San Diego, CA, USA). A probability value of *p* < 0.05 was considered to be statistical significant.

## 4. Conclusions

In the present study the seeds of two wild grown *Sambucus* (*nigra* and *ebulus*) species from Romania (Transylvania) were analyzed with respect to oil yields and fatty acid contents of total lipids and corresponding fractions (neutral and polar). The positional distribution of fatty acids in seed TAGs were also determined to obtain detailed data to assess the chemical and nutritional properties of *Sambucus* seed oils and theirs potential for human consumption, as alternatives to the conventional vegetable oils.

This work demonstrated that *Sambucus* seeds could be considered rich sources of oil (more than 22 g oil/100 g seeds). The oil TAGs were similar in fatty acid composition to the TLs, containing substantial amounts of α-linolenic, linoleic and oleic acids. The PL fractions and all the minor NL subclasses (MAGs, DAGs, FFAs and SEs) were also highly unsaturated. A clear characteristic of the SEs and FFAs were the significantly high levels of SFAs (over 18%), with considerable amounts of palmitic and stearic acids. *Sambucus* seed oils, with their high levels of α-linolenic acid together with a near 1:1 ratio of n-6 to n-3 PUFAs represent very balanced sources of essential PUFAs for human health. This conclusion is also supported by the results of the positional analysis of fatty acids in TAGs.

## Supplementary Materials

Supplementary File 1
