# Supplementary figures and images for: Lipid Classes and Fatty Acid Regiodistribution in Triacylglycerols of Seed Oils of Two Sambucus Species (S. nigra L. and S. ebulus L.)
Source: Molecules. 2013 Sep 25;18(10):11768–82. doi: 10.3390/molecules181011768 (PMC6269702; doi:10.3390/molecules181011768)

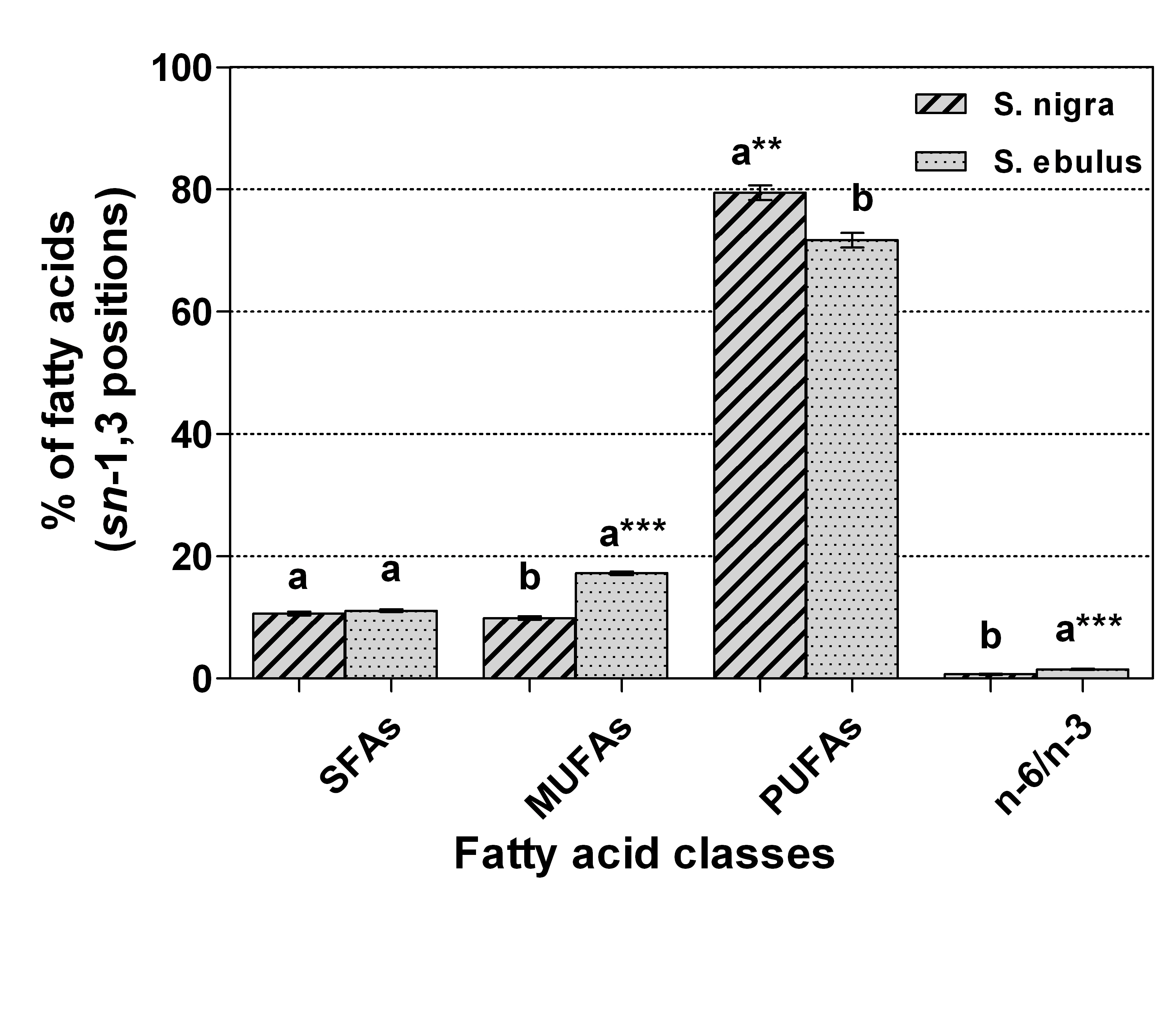

Supplement: Supplementary File 1 [file molecules-18-11768-s001.zip › Fig 2 art SAMBUCUS.jpg]

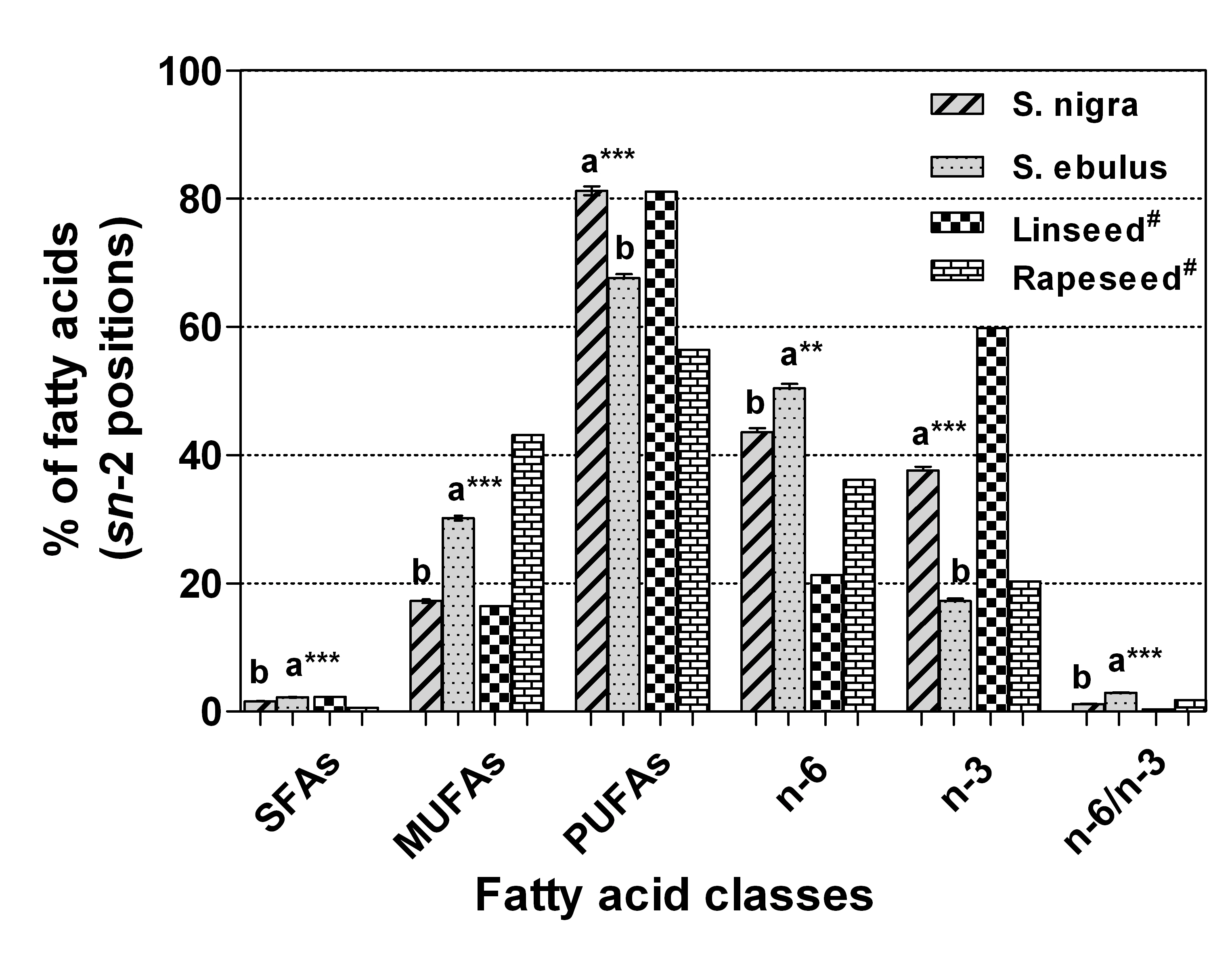

Supplement: Supplementary File 1 [file molecules-18-11768-s001.zip › Fig 3 sambucus.jpg]
